# Vaccination using mutated receptor binding domains of SARS-CoV-2: Evidence for partial immune escape but not serotype formation

**DOI:** 10.3389/fimmu.2023.1114396

**Published:** 2023-02-10

**Authors:** Xinyue Chang, Xuelan Liu, Byron Martina, Andris Zeltins, Gilles Augusto, Monique Vogel, Mona O. Mohsen, Daniel E. Speiser, Martin F. Bachmann

**Affiliations:** ^1^ International Immunology Centre, Anhui Agricultural University, Hefei, China; ^2^ Department of Rheumatology and Immunology, University Hospital Bern, Bern, Switzerland; ^3^ Department of BioMedical Research, University of Bern, Bern, Switzerland; ^4^ Artemis Bio-Support, Delft, Netherlands; ^5^ Latvian Biomedical Research and Study Center, University of Riga, Riga, Latvia; ^6^ Jenner Institute, University of Oxford, Oxford, United Kingdom; ^7^ Saiba GmbH, Pfäffikon, Switzerland

**Keywords:** SARS–CoV–2, variants, serotype, immune escape, antibody

## Abstract

**Introduction:**

SARS-CoV-2 has developed a number of Variants of Concern (VOC) with increased infectivity and/or reduced recognition by neutralizing antibodies specific for the receptor binding domain (RBD) of the spike protein. Extended studies of other viruses have shown that strong and broad viral escape from neutralizing serum antibodies is typically associated with the formation of serotypes.

**Methods:**

To address the question of serotype formation for SARS-CoV-2 in detail, we generated recombinant RBDs of VOCs and displayed them on virus-like particles (VLPs) for vaccination and specific antibody responses.

**Results:**

As expected, mice immunized with wild type (wt) RBD generated antibodies that recognized wt RBD well but displayed reduced binding to VOC RBDs, in particular those with the E484K mutation. Unexpectedly, however, antibodies induced by the VOC vaccines typically recognized best the wt RBDs, often more than the homologous VOC RBDs used for immunization. Hence, these data do not reveal different serotypes but represent a newly observed viral evolution, suggesting a unique situation where inherent differences of RBDs are responsible for induction of neutralizing antibodies.

**Discussion:**

Therefore, besides antibody (fine) specificity, other qualities of antibodies (e.g. their affinity) determine neutralizing capability. Immune escape of SARS-CoV-2 VOCs only affects a fraction of an individual’s serum antibodies. Consequently, many neutralizing serum antibodies are cross-reactive and thus protective against multiple current and future VOCs. Besides considering variant sequences for next generation vaccines, broader protection will be achieved with vaccines that induce elevated titers of high-quality antibodies.

## Introduction

The interaction between the receptor binding domain (RBD) of the spike protein of SARS-CoV-2 and the angiotensin converting enzyme (ACE2) receptor is the key to viral infection and therefore to vaccine design aiming at induction of neutralizing antibodies. Numerous studies have demonstrated that RBD-specific antibodies are able to block RBD from binding to ACE2 resulting in neutralization of SARS-CoV-2. However, the emerged variants of concern (VOCs) have evolved their RBDs to increase the binding affinity to ACE2 (1–4), which is one of the strategies of viral escape and higher transmission rate. For instance, Rajah et al. demonstrated higher affinity to ACE2 of the Alpha (B.1.1.7) and Beta (B.1.351) spike proteins, and better syncytia formation than the ancestral D614G strain (5). Interestingly, the Delta (B.1.617.2) spike protein was found to fuse faster, which may be an explanation for its high transmissibility (6). More recently, Bowen et al. determined binding to ACE2 of Omicron variants and showed higher affinity for BA.1/BA.2, and particularly for BA.4/BA.5 (4).

Besides increased affinity of RBD to ACE2, the SARS-CoV-2 VOCs show reduced recognition by antibodies (7). It was shown that B.1.351 strain was refractory to several monoclonal antibodies targeting RBM (receptor binding motif) and resistant to neutralization by convalescent plasma and vaccinated sera (8). An independent study demonstrated that B.1.351 and P.1 variants were resistant to treatment by monoclonal antibodies and escaped from antibodies induced by infection and vaccination (9). The neutralization titers of the Omicron variant B.1.1.529 by BNT162b2 vaccinated human sera (two doses) were >22-fold lower than Wuhan strain (10). Neutralizing antibody 58G6, isolated from a convalescent patient, was found to recognize AA450-458 and 470-495 regions on RBD. Although it is able to neutralize Omicron BA.1, its efficacy is 40-fold lower than wt neutralization (11). In addition to E484K, K417N is shown to abrogate RBD binding of many neutralizing antibodies (12). Unfortunately, recent variants escape broadly, to the extent that BQ.1.1 could not be neutralized by any of the therapeutic monoclonal antibodies tested (13).

Interestingly, escape from neutralizing antibodies may be caused not only by epitope changes, but also through increased affinity of virus-receptor binding that may outcompete antibody binding. Indeed, we have recently demonstrated that increased binding of RBD to ACE2 not only results in enhanced infection but also reduced neutralization by RBD-specific antibodies because of their diminished capability to outcompete RBD-ACE2 binding, a phenomenon termed affinity escape (1, 14).

The viral escape owing to either increased affinity to its receptor or reduced recognition by antibodies has raised the question of a need for (yearly) adaptation of COVID-19 vaccines as is known for flu vaccines (15). In particular, VOCs with the E484K mutation in RBD escape recognition by many of the antibodies induced by wt SARS-CoV-2 infection (16). This raises the possibility of emerging serotypes, i.e. viral strains that induce antibodies that do not cross-react at the level of neutralization. Well-known viral examples for serotype formation are Polio (3 serotypes) as well as Dengue (4 serotypes) viruses in humans. In research, the vesicular stomatitis virus (VSV) serotypes “Indiana” and “New Jersey” represent one of the best studied viral serotype models in mice (17). An essential part of serotype definition is mutual symmetry, i.e. neutralizing antibodies against serotype I do not cross-neutralize serotype II, and neutralizing antibodies against serotype II do not cross-neutralize serotype I. Therefore, vaccination programs against those “serotype-forming” viruses usually should target all serotypes as is the case for Polio and strongly aspired for Dengue (18, 19).

Whether SARS-CoV-2 form serotypes still remains unknown, which is of great importance to vaccination programs globally. To address this question experimentally, we generated RBDs with emerged key mutations and displayed them on CuMV_TT_, an immunologically optimized virus-like particles (VLPs) derived from Cucumber Mosaic Virus packaging bacterial RNA as toll-like receptor 7/8 ligand as well as containing a universal T cell epitope derived from tetanus toxin (20). This T cell epitope stimulates T helper cells efficiently, which is particularly useful in elderly and results in enhanced antibody responses (21). We found that the antibodies generated by wt vaccine recognized and neutralized best the wt virus. Interestingly, vaccines derived from VOC RBDs induced antibodies recognizing well the wt RBD, often even better than the VOC RBD used for immunization. Consistently, the titers showed the same hierarchy of variant neutralization, irrespective of the vaccine used to immunize the mice, with best neutralization of wt virus, followed by Beta, Delta and Gamma viruses, and lowest neutralization of Omicron virus. These findings demonstrate that SARS-CoV-2 virus has not formed serotypes despite it may escape from neutralizing antibodies.

Our results are compatible with several studies showing that variant vaccines are not necessarily better for the induction of neutralizing antibodies against the homologous variant virus (22). Wt-RBD remains an important pillar of current vaccines, even for protection from VOCs. Thus, for further vaccine development, the integration of novel mutations may not be the predominant goal. Probably, vaccine efficacy depends on inducing high quantities of efficient antibodies, including high affinity antibodies that are cross-reactive and can successfully compete with the increased ACE2 affinity of VOCs (1, 14).

## Materials and methods

### Animals

*BALB/cOlaHsd* mice were purchased from from Envigo (Horst, The Netherlands) at the age of 7 weeks. Mice were kept in the specific pathogen-free (SPF) facility in the Department of BioMedical Research of the University of Bern, Switzerland. All studies involving animals were approved by the Cantonal Veterinary Office in Bern. All animal experiments were performed in accordance with the regulations and guidelines of the Cantonal Veterinary Office in Bern, Switzerland (license Nr. BE70/18).

*BALB/cOlaHsd* female mice (8-12 weeks) were immunized with 40 μg (60 μl) CuMV_TT_ VLP-based vaccines subcutaneously at day 0 and day 28. Serum samples were collected weekly from day 14 until 49. Five mice were used for every immunization group. All mice used in experiments did not show body weight loss or other observative harmful signs until day 49, when they were euthanized.

### Production of vaccines

The RBDs of wt and VOCs (RBD_417_, RBD_484_, RBD_501_, and RBD_trip_) were produced as described previously (23). Briefly, plasmids containing the RBD-encoding genes were transfected into Expi293F cells using ExpiFectamine 293 transfection kit (Gibco, Thermo Fisher Scientific, Waltham, MA, USA) according to the manufacturer’s instruction. Cell supernatants containing RBD proteins were collected and filtered through 0.2 μm filter, which subsequently were purified with HiTrap TALON crude column (Cytiva, Marlborough, MA, USA) in Äkta pure system. The eluted RBDs were buffer exchanged with PBS and concentrated in Vivaspin centrifuge concentrator (MWCO 5kD, Sartorius, Goettingen, Germany). BCA assay (Thermo Fisher Scientific, Waltham, MA, USA) was used to determine purified RBDs concentration. Purity of RBDs was analyzed by SDS-PAGE gel.

The CuMV_TT_ VLPs were expressed in *E.coli* and purified using sucrose gradient ultracentrifugation, as described previously (24). The RBDs were conjugated to CuMV_TT_ VLPs *via* the cross-linker Succinimidyl 6-(beta-maleimidopropionamido) hexanoate (SMPH) (Thermo Fisher Scientific, Waltham, MA, USA). Basically, the RBDs were first exchanged to VLP storage buffer (5 mM Phosphate buffer with 2 mM EDTA, pH 7.2) to prevent VLP disassembly after coupling. the VLP was firstly reacted with SMPH at a molarity ratio of SMPH: RBD = 5:1 at 25˚C, 400 rpm shaking for 30 min. Then the SMPH-linked VLPs were incubated with RBD at 25˚C, 400 rpm shaking for 3 hours, which was reduced by Tris-(2-Carboxyethyl) phosphine (TCEP) (Invitrogen, Waltham, MA, USA). The molarity ratio of VLP to RBD was optimized to 2:1. The coupling efficiency was analyzed in SDS-PAGE gel by densitometry. All RBDs demonstrated the efficiency of 20-30%. Furthermore, the contents of prokaryotic ssRNA loaded from *E.coli* were checked in agarose gel. The vaccines used for immunization were stored in 5 mM Phosphate buffer with 2 mM EDTA, pH 7.2. The concentration was calculated based on VLP concentration (0.67 mg/ml).

### Transmission electron microscope

The CuMV_TT_-based vaccines were assessed by TEM for intact viral shape. 5 μl of samples was pipetted to grids, and then the grids were rinsed by dipping into ddH_2_O for 3 times. Samples were stained for 45 s with 2% uranyl acetate solution (Electron Microscopy Science, Hatfield, PA, USA), and excess staining solution was removed by filter paper. Afterwards, images were captured with a digital camera (Veleta, Olympus, Münster, Germany) under a transmission electron microscope (Tecnal Spirit, FEI, Hillsboro, OR, USA) at 80 kV.

### ELISA

RBD proteins were coated at 1 μg/ml concentration in half area Corning 96-well plates at 4˚C overnight. Plates were washed with PBS for 4 times and blocked with PBS-0.15% Casein at room temperature for 2 hours, after which serum samples were added and incubated for one hour. Afterwards, detection antibody goat anti-mouse IgG-HRP (Jackson ImmunoResearch, West Grove, PA, USA) was added and incubated for one hour. The detection antibody was washed away by 4 times PBS-0.01% Tween. Finally, plates were developed with TMB solution in citrate buffer and stopped with 1M H_2_SO_4_ solution. Plates were read at OD_450nm_ and antibody titers were determined as the serum dilution times to reach half of the OD_max_ value (OD_50_).

IgA antibody responses were assessed in a similar way with one more step to process the serum samples. To avoid underestimating the IgA responses, IgG antibodies were removed from serum by incubating with Protein G magnetic beads (Thermo Scientific, Waltham, MA, USA) for 10 min at room temperature. Goat anti-mouse IgA-HRP antibody (ICN Cappel, Costa Mesa, CA, USA) was used for IgA detection.

### Avidity ELISA

The quality of IgG antibodies was measured by avidity ELISA. In this assay, two plates were performed in parallel until adding the detection antibody. One plate was washed with 7 M urea in PBS-0.05% Tween and the other only with PBS-0.05% Tween for 3 times, each time 5 min incubation. The antibodies remaining after urea wash are regarded as high-avidity antibodies. The titers of total and high-avidity IgG antibodies were displayed.

### Neutralization assay

We prepared SARS-CoV-2 wild type (wt, Wuhan) virus and VOCs with RBD mutations as follows: K417N, E484K, N501Y (Beta), K417T, E484K, N501Y (Gamma), L452R, T478K, E484K (Delta), and G339D, S371L, S373P, S375F, K417N, N440K, G446S, S477N, T478K, E484A, Q493K, G496S, Q498R, N501Y, Y505H (Omicron). These viruses were tested for neutralization by the serum samples from immunized mice. Briefly, heat-inactivated (56˚C for 30 min) serum was diluted at 2-fold serial dilution from 1:20 to 1:160. Then, 100 TCID_50_ of SARS-CoV-2 (variant) virus were added to each diluted serum sample and incubated for 1 hour at 37˚C. As read-out, the presence of cytopathic effect (CPE) in each well was observed and neutralization titers were displayed as the highest dilution times of serum that fully inhibits 100% CPE formation.

## Results

### Generation and characterization of CuMV_TT_-based vaccines against SARS-CoV-2

**Figure 1:**
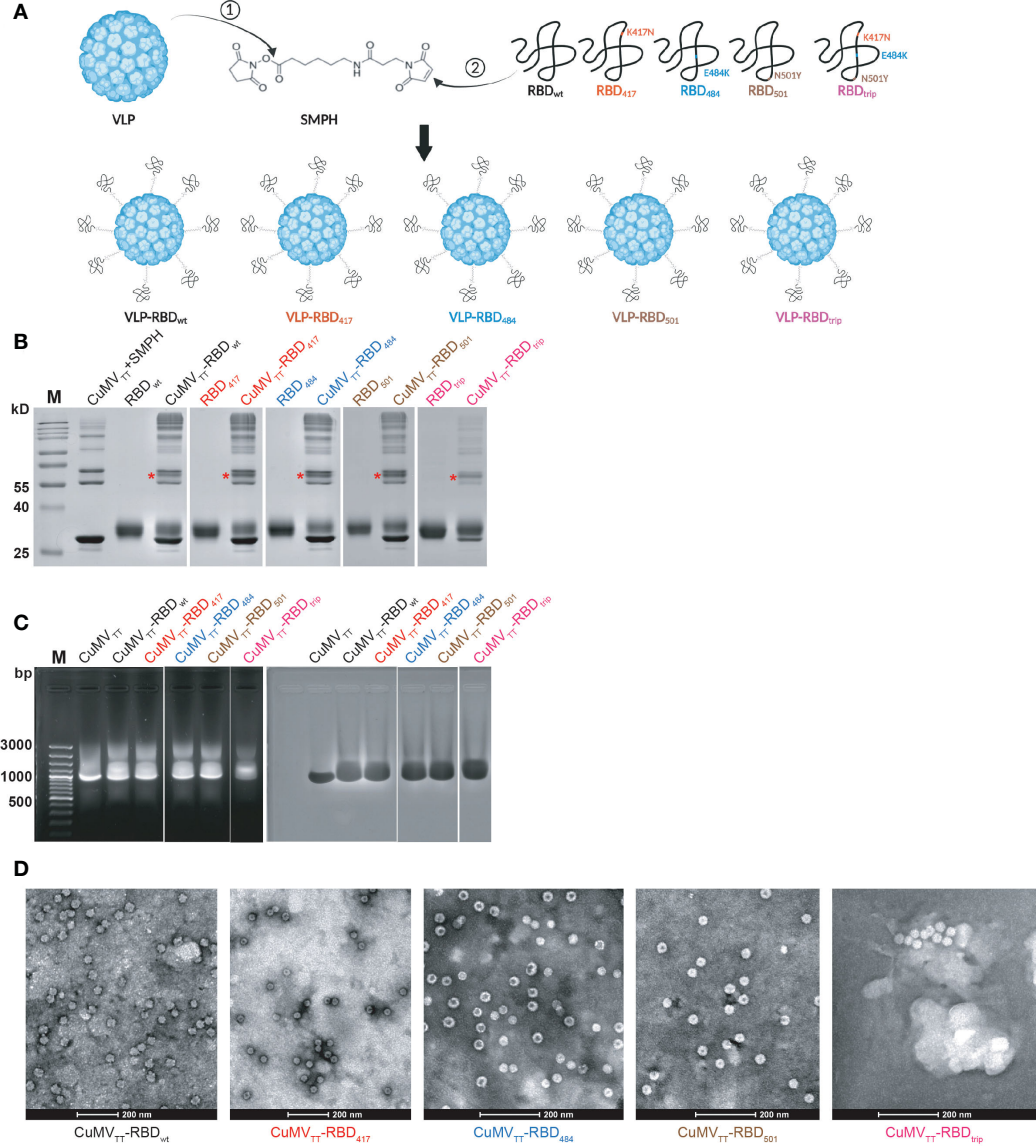
CuMV_TT_-based vaccines were generated by covalently linking RBD_wt_, RBD_417_, RBD_484_, RBD_501_, and RBD_trip_ to CuMV_TT_ *via* SMPH **(A)**; CuMV_TT_-based vaccines were examined in SDS-PAGE gel to confirm the coupling efficiency **(B)**, agarose gel for RNA content **(C)**, transmission electron microscopy for intact viral shape **(D)**. The monovalent coupling was indicated by *.

We have demonstrated previously that the VLP vaccine CuMV_TT_-RBD_wt_ is highly immunogenic and induces high IgG titers against RBD_wt_ and RBD of VOCs, able to neutralize wt Wuhan strain and VOCs (23, 24). Using the same strategy, we generated CuMV_TT_-based variant vaccines, including RBDs with mutations that have been described to alter antibody recognition. Accordingly, we produced RBDs with the mutations K417N (RBD_417_), E484K (RBD_484_), N501Y (RBD_501_) and all three K417N, E484K, N501Y mutations (RBD_trip_) (**Figure 1A**). Please note that we failed to express RBD from Omicron variants in sufficient quantities for vaccine candidate generation. All vaccines showed similar coupling efficiencies (**Figure 1B**), contained comparable amounts of RNA (**Figure 1C**) and maintained viral shape (**Figure 1D**).

### Immunogenicity of VOC RBD vaccines

**Figure 2:**
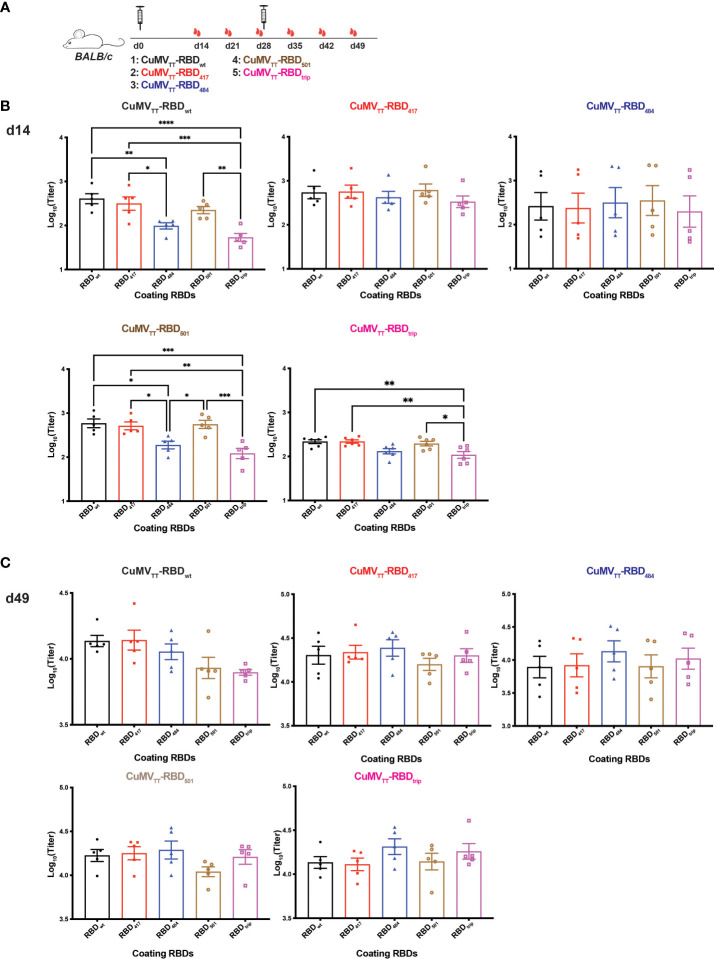
Immunogenicity of vaccines derived from VOC RBDs. **(A)** BALB/c mice were immunized on d0 and d28 with CuMV_TT_-RBD_wt_, CuMV_TT_-RBD_417_, CuMV_TT_-RBD_484_, CuMV_TT_-RBD_501_, and CuMV_TT_-RBD_trip_, and serum samples were collected on d14, 21, 28, 35, 42, 49. IgG antibody titers against RBD_wt_, RBD_417_, RBD_484_, RBD_501_, and RBD_trip_ induced by all vaccines on d14 **(B)** and d49 **(C)**. One-way ANOVA analysis was performed in Prism 9 (n=5), α=0.5 and statistical significance was displayed as p ≤ 0.05 (*), p ≤ 0.001 (**), p ≤ 0.005 (***), p ≤ 0.001 (****).

To test immunogenicity of the vaccine candidates, mice were immunized on day 0 (d0) and d28 and sera were collected before (d14, 21, 28) and after (d35, 42, 49) booster (**Figure 2A**). Sera of the different mice were subsequently tested by ELISA. As found previously, mice immunized with CuMV_TT_-RBD_wt_ induced strong responses against RBD_wt_. Vaccines derived from RBDs of variants also demonstrated potent immunogenicity. However, recognition of some of the mutated RBDs was less efficient, with RBDs containing the E484K mutation being particularly poorly recognized (**Figure 2B**). These differences were more pronounced after priming than after booster (**Figure 2C**). Another study using prokaryotic cells producing RBDs showed that RBD containing E484K mutation was more immunogenic after three immunizations together with Freund’s adjuvants (25), which is not comparable with our results because the lack of glycosylation changes RBD conformation, and repeated immunization may shield primary differences. Interestingly, mice immunized with CuMV_TT_-RBD_trip_ generated antibodies that recognized wt RBD better than RBDs with the E484K mutation. Hence, unlike expected for serotypes where each variant vaccine induces best responses to its homologous RBD (symmetrically), recognition was asymmetric with equal or even better recognition of wt RBD than variant RBDs, particularly E484K mutated RBDs. Hence, this asymmetry is incompatible with formation of new serotypes by VOCs.

**Figure 3:**
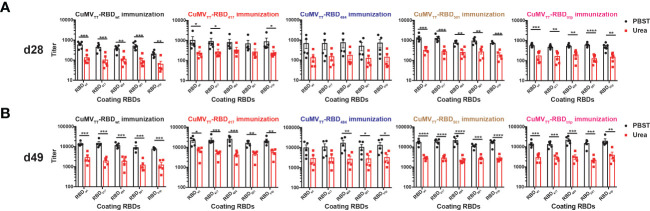
IgG antibody titers of avidity ELISA on d28 **(A)** and d49 **(B)**. Unpaired t-test was performed in Prism 9 (n=5), α=0.5 and statistical significance was displayed as p ≤ 0.05 (*), p ≤ 0.001 (**), p ≤ 0.005 (***), p ≤ 0.001 (****).

Interestingly, avidity measurements failed to indicate increased avidity of antibodies induced by VOC-RBDs against the respective VOCs compared to the other viruses (**Figure 3**).

**Figure 4:**
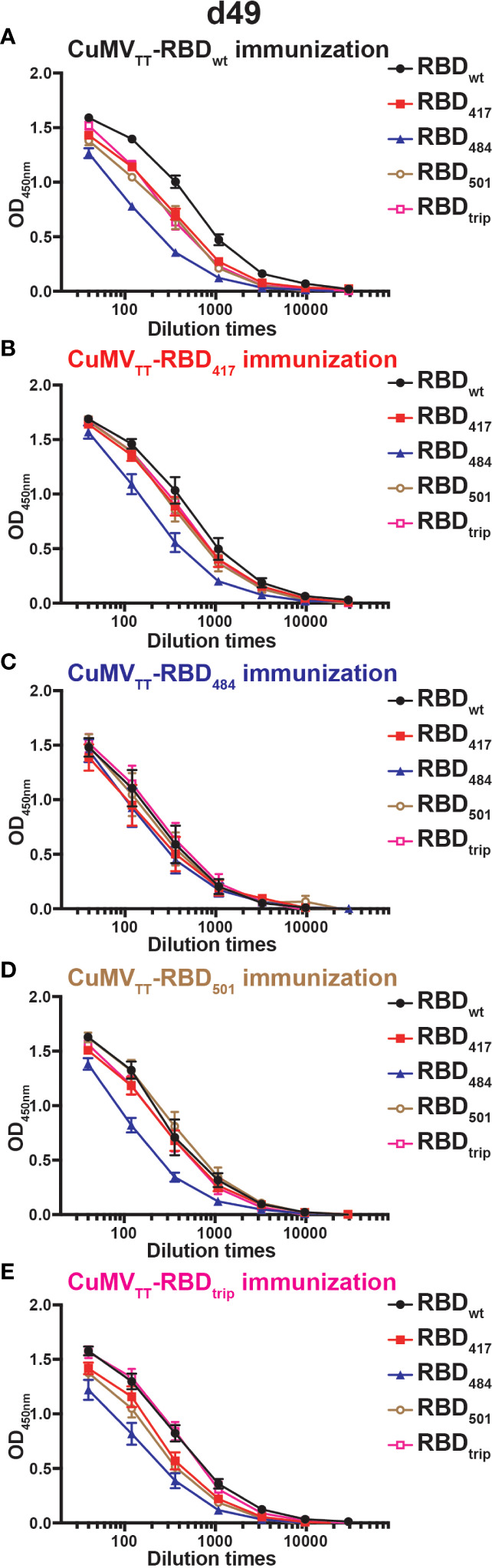
IgA antibody responses of mice sera on d49 immunized with CuMV_TT_-RBD_wt_ **(A)**, CuMV_TT_-RBD_417_ **(B)**, CuMV_TT_-RBD_484_ **(C)**, CuMV_TT_-RBD_501_ **(D)**, or CuMV_TT_-RBD_trip_ **(E)**. ELISA curves of OD values with serum dilution were displayed.

We also searched for the presence of IgA and found that all vaccine candidates induced strong IgA responses in the serum, which is reassuring given that IgA is required for mucosal immunity. As observed for IgG, wt RBD induced good IgA responses against all RBDs, with the exception of RBDs containing the E484K mutation which were less well recognized. This pattern applied to antibodies induced by all RBDs, including those containing the E484K mutation, again indicating that RBDs differ in their inherent ability to induce and be recognized by antibodies and do not show symmetric recognition patterns as known for serotypes (**Figures 4A–E**).

### Neutralization of variant viruses by vaccine-elicited antibodies

**Figure 5:**
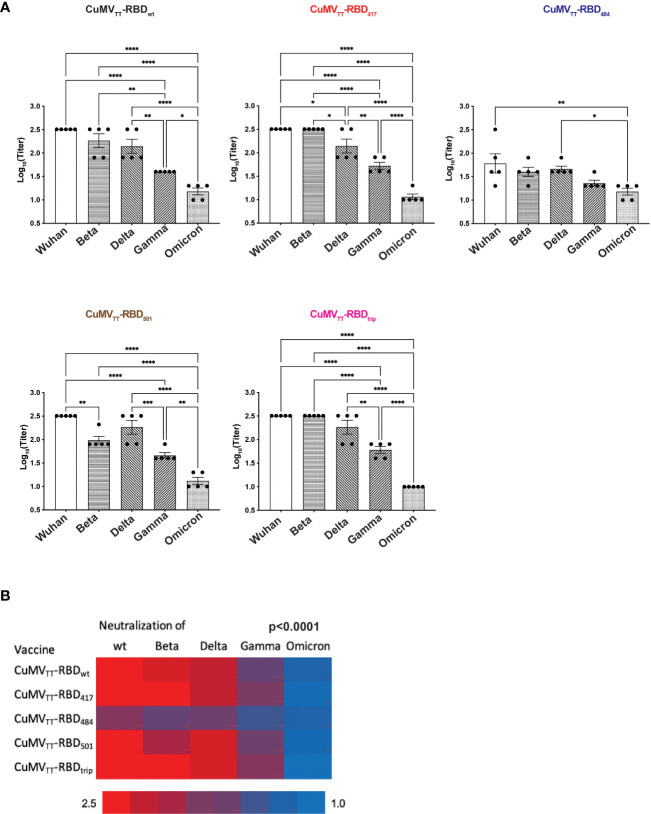
Neutralization of wt Wuhan and Beta, Delta, Gamma and Omicron variants by d49 sera. **(A)** Titers were expressed as the sera dilution times to form 100% CPE. One-way ANOVA analysis was performed in Prism 9 (n=5), α=0.5 and statistical significance was displayed as p ≤ 0.05 (*), p ≤ 0.001 (**), p ≤ 0.005 (***), p ≤ 0.001 (****). **(B)** Heatmap of neutralization titers of wt and VOCs by immunized sera, Chi2-test was performed.

Protection from infection and severe disease depends on the induction of neutralizing antibodies. Interestingly, the pattern of the induced neutralizing antibodies was very similar to the observations shown above for antibody recognition (**Figure 5A**). Vaccination with wt RBD induced neutralizing antibody responses against all VOCs, whereas neutralization was clearly inferior for strains containing the E484K mutation. Interestingly, in an asymmetric fashion, antibodies induced by vaccines containing the E484K mutation neutralized wt Wuhan virus better than or equal to VOC RBDs containing the E484K mutation.

For systematic comparison of vaccine efficacy, we quantified neutralization of wt strain and VOCs. Strikingly, none of the variant vaccines induced superior neutralization of their homologous VOC compared with wt virus (**Figure 5B**). On the contrary, antibodies induced by the VOC vaccines showed better neutralization of the wt strain than the homologous VOC used for immunization. This assessment shows in a statistically highly significant manner (p<0.0001) that the use of variant vaccines did not lead to symmetrically optimized protection against homologous VOCs, stressing the asymmetry between virus and neutralizing antibodies.

Taken together, our data formally demonstrate that the known VOCs of SARS-CoV-2 are not constituting new serotypes but reflect an adaptation of the virus to the human population and its neutralizing antibodies without classical serotype formation. These considerations have important implications for future vaccine strategies, as newly adapted vaccines appear less of an issue than broad access to vaccines and maintenance of highly efficient antibodies at high levels which is optimal for neutralization even of variants with mutations that reduce the binding of some antibodies.

## Discussion

Following the successful eradication of Poliovirus serotype 2 (with exception of outbreak in 2019), vaccination was done with the bivalent oral poliovirus vaccine containing serotype 1 and 3 (18, 26). Recently, the poliovirus type 3 was announced to be eradicated globally (27). Would such a strategy also work for SARS-CoV-2 VOCs? To answer this question, it is necessary to clarify whether SARS-CoV-2 has generated serotypes. Here we conjugate RBDs from VOCs to VLPs and determined the antibody responses after immunizing mice. Our results indicate that the early VOCs (Beta, Gamma, and Delta) do not show serotype fashion. For example, the E484K containing RBD vaccines elicited antibodies that neutralized RBD_wt_ better than RBDs from VOCs.

Due to the massive mutations in spike protein of Omicron variant and reduced antibody cross-reactivity, it has been proposed that Omicron variants may form a second serotype distinct from other ancestral variants (28). This hypothesis is consistent with our conclusion that the Beta, Gamma, and Delta do not form serotypes. However, recent data indicate the contrary. For instance, Reynolds et al. demonstrated that infection of Omicron variant reduced the magnitude of neutralizing antibodies and T cell responses against itself while it enhanced responses against other VOCs in 3-dose fully vaccinated individuals, confirming that immunization with a particular strain might not always trigger a potent immune response against that strain (29). Additionally, new Omicron subvariants BA.2.12.1, BA.4, BA.5 escape the immunity elicited by BA.1 infection (30). Thus, it remains uncertain whether the Omicron variants form a second serotype, and more evidence is required on this topic. Unfortunately, we are unable to generate a VLP-based vaccine particularly for Omicron in this work, while we are currently working on it.

Another opinion is even to rename the Omicron variants as SARS-CoV-3 due to the drastic immune evasion (31), which was however argued to be contrary to nomenclature standards (32). The genome differences between coronaviruses SARS-CoV-2, SARS-CoV, MERS-CoV and Omicron variants BA.2, BA.2.12.1, BA.4 and BA.5 were analyzed, and found insufficient for defining a novel virus (33). In addition, the cross-recognition of antibody induced by distinct viral sublineages was limited, but still not revealing a new virus. In conclusion, Omicron variants clearly demonstrated differences regarding mutation and partial immune evasion from earlier variants, yet whether they are qualified for new serotype or new name is still under discussion.

The finding that neutralization titers of Omicron virus were relatively lower than other VOCs is consistent with previous studies, which is one of the reasons why Omicron virus frequently escape from vaccinated individuals (10, 34). To improve the vaccine efficacy against Omicron variants, recent vaccine development studies target Omicron variants. Interestingly, Gagne et al. demonstrated that a booster of wt vaccine or Omicron vaccine induced comparable neutralization capacity against Omicron virus challenge in non-human primates after 4 weeks (35). A similar study showed that boosting with either wt vaccine or Omicron vaccine protect mice from Omicron virus infection (36). Furthermore, memory B cells were activated to generate antibodies that neutralize partial Omicron variant after a third dose booster with wt vaccine (37). These studies may imply that a booster with current wt vaccine instead of a new variant-targeting vaccine is demanded for Omicron variants (25,). The partial protection against Omicron of vaccinated individuals may be attributed to the remaining neutralizing antibodies and recall of the mature, high-affinity memory B cells to produce neutralizing antibodies recognizing conserved RBD regions (22). However, this protection decays with the constant evolution of virus, in which case a new vaccine targeting to the new variant will be needed.

In general, a vaccine against the current circulating variant is the most efficient tool to prevent a pandemic. Moderna announced that a booster with a new bivalent vaccine based on wt and Omicron BA.1 stimulated stronger antibody responses against BA.1, compared with wt booster. Nevertheless, the efficacy against BA.4 and BA.5 of BA.1 booster dropped 2-3 folds (38). The fast-evolving virus complicates the decision to keep the original vaccine or update upon new variants, taking consideration of new vaccine manufacturing and registration.

In contrast to the considerable focus on variants in R&D of COVID-19 vaccines, there is only relatively little emphasis on the quality of vaccine-induced antibodies. Yet, protection against SARS-CoV-2 likely depends on large quantities of antibodies with sufficiently high affinity (1, 14). The fact that SARS-CoV-2 is not readily forming new serotypes, together with the observation that high affinity antibodies are capable to overcome viral escape mutations by virtue of their cross-reactivity, further support the notion that antibody affinity deserves attention for the development of novel vaccines. This appears also promising for protection against future variants. As long as viral mutations continue to escape only some but not all of the antibodies of a vaccinated individual, there is good reason to improve the responses of those B cells that produce broadly and efficiently neutralizing high affinity antibodies potentially capable to protect against many (current and future) variants, a priority aim in vaccine development.

It is unlikely that antibodies make coronavirus infections worse in humans. In contrast to Dengue and Zika viruses, antibody-dependent enhancement (ADE) was not observed in SARS-CoV-2 infection (39). ADE is generally very unlikely, because the target cells of SARS-CoV-2 are non-immune cells that do not express Fcγ receptors which mediate the viral uptake responsible for ADE (40).

Although there is clear evidence that T cells also contribute to cross-protection (41), it remains likely that neutralizing antibodies are of central importance, given the facts that the efficacy of vaccines generally relies on neutralizing antibodies, and that insufficient generation of neutralizing antibodies is the dominant cause for vaccine failure (42, 43).

In conclusion, the broad spectrum of immunity elicited by vaccine should be the aim for future vaccine strategy, for which the serological information of variants is required. Our study using vaccines based on CuMV_TT_-VLPs indicate that the variants before Omicron do not form serotype. The situation for consistently mutating Omicron variants is uncertain, however, vaccines targeting Omicron are not the terminators of the COVID-19 pandemic. Practically, our findings may explain why WT RBD is included in newer booster vaccines against Omicron variants, despite that no scientific explanation has been provided so far. While Omicron variants have acquired multiple mutations that contribute to infectivity and immune escape, the viral structure still remains relatively conserved such that protection is still possible through neutralizing antibodies elicited by previous variants and vaccines. Boosting such neutralizing antibodies may be optimized by promoting increased quality responses, particularly of high affinity antibodies that are enriched by affinity maturation (44).

## Data availability statement

The original contributions presented in the study are included in the article/**Supplementary Material**. Further inquiries can be directed to the corresponding author.

## Ethics statement

The animal study was reviewed and approved by Cantonal Veterinary Office in Bern.

## Author contributions

Conceptualization: MV, DS, MB. Methodology: XC, XL, BM, AZ, GA, MM. Investigation: XC, XL, BM, AZ. Visualization: XC, XL. Funding acquisition: MV, MB. Project administration: XC, XL. Supervision: MV, MM, DS, MB. Writing–original draft: XC, DS, MB. Writing–review and editing: XC, MV, MM, DS, MB. All authors contributed to the article and approved the submitted version.

## Supplementary material

Supplementary Figure 1 IgG antibody titers on d21 **(A)** and d28 **(B)** against RBDs. One-way ANOVA analysis was performed in Prism 9 (n=5), α=0.5 and statistical significance was displayed as p ≤ 0.05 (*), p ≤ 0.001 (**), p ≤ 0.005 (***), p ≤ 0.001 (****).

Supplementary Figure 2 IgG antibody titers on d35 **(A)** and d42 **(B)** against RBDs. One-way ANOVA analysis was performed in Prism 9 (n=5), α=0.5 and statistical significance was displayed as p ≤ 0.05 (*).
