# Supplementary figures and images for: Vaccination using mutated receptor binding domains of SARS-CoV-2: Evidence for partial immune escape but not serotype formation
Source: Front Immunol. 2023 Feb 10;14:1114396. doi: 10.3389/fimmu.2023.1114396 (PMC9950740; doi:10.3389/fimmu.2023.1114396)

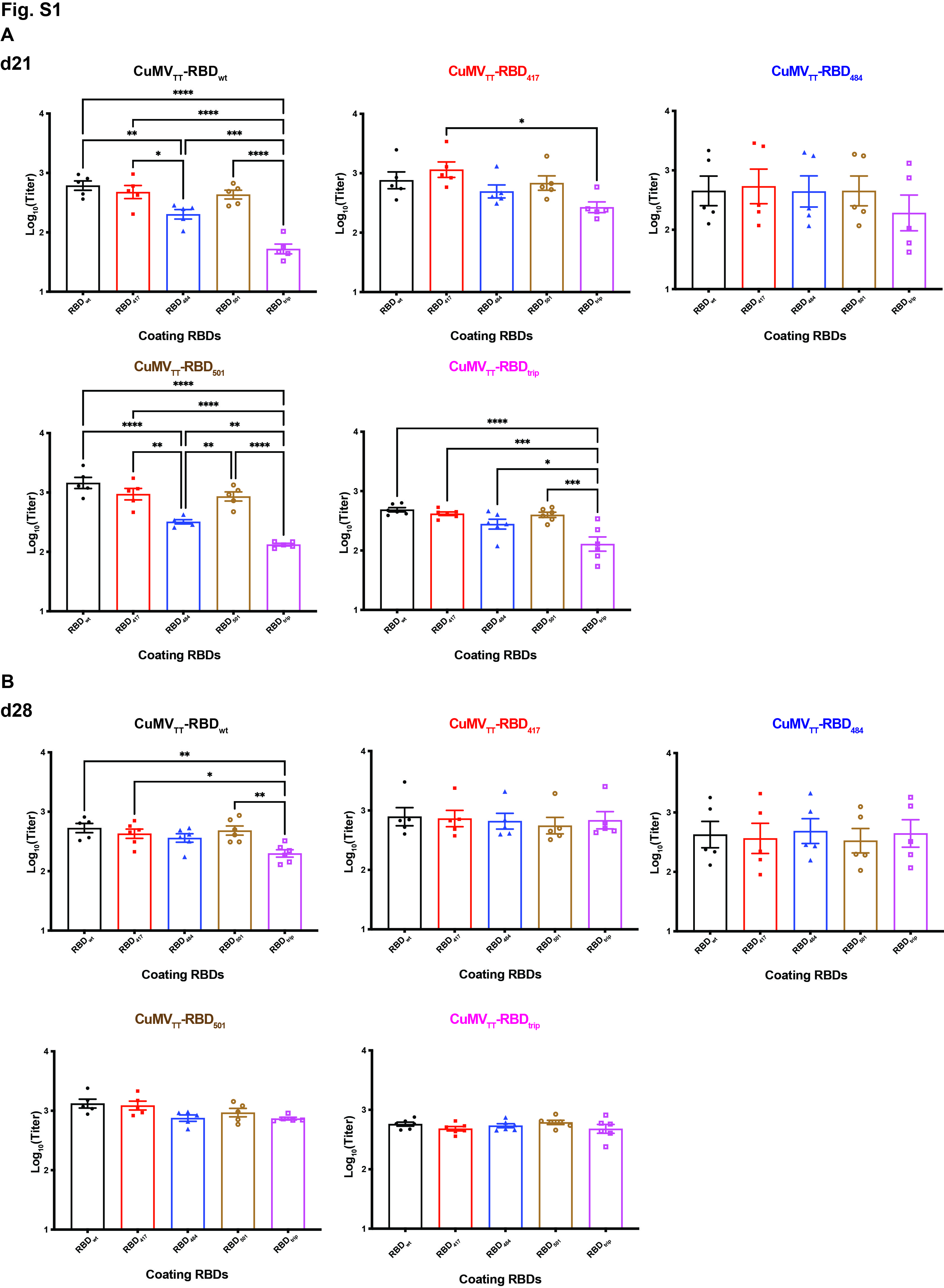

Supplement: Supplementary Figure 1 — IgG antibody titers on d21 (A) and d28 (B) against RBDs. One-way ANOVA analysis was performed in Prism 9 (n=5), α=0.5 and statistical significance was displayed as p ≤ 0.05 (*), p ≤ 0.001 (**), p ≤ 0.005 (***), p ≤ 0.001 (****). [file Image_1.jpeg]

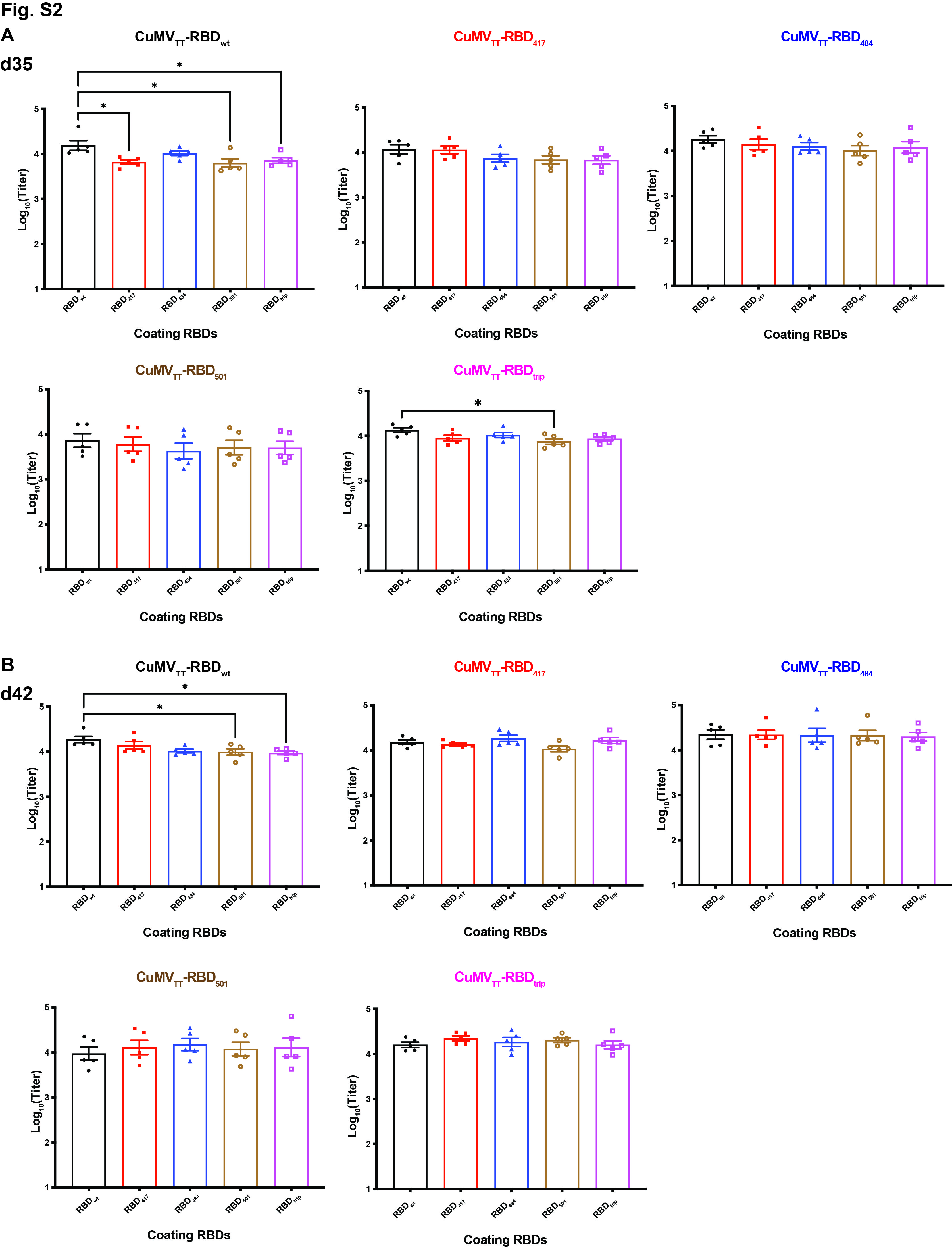

Supplement: Supplementary Figure 2 — IgG antibody titers on d35 (A) and d42 (B) against RBDs. One-way ANOVA analysis was performed in Prism 9 (n=5), α=0.5 and statistical significance was displayed as p ≤ 0.05 (*). [file Image_2.jpeg]
